# Testosterone Inhibits Secretion of the Pro-Inflammatory Chemokine CXCL1 from Astrocytes

**DOI:** 10.3390/cimb46030135

**Published:** 2024-03-06

**Authors:** Malgorzata Turniak-Kusy, Maciej Studzian, Piotr Szpakowski, Piotr Kuchta, Kaja Smietanka, Claudia Mattern, Lukasz Pulaski, Bartosz Bielecki

**Affiliations:** 1 Department of Neurology and Stroke, Medical University of Lodz, 90-549 Lodz, Poland; 2 Department of Oncobiology and Epigenetics, Faculty of Biology and Environmental Protection, University of Lodz, 90-237 Lodz, Poland; 3 Laboratory of Transcriptional Regulation, Institute of Medical Biology, Polish Academy of Sciences, 90-364 Lodz, Poland; 4 Faculty of Medicine, Medical University of Lodz, 90-419 Lodz, Poland; 5 Oceanographic Center, Nova Southeastern University, Fort Lauderdale, FL 33314, USA; 6 M&P Pharma AG, 6376 Emmetten, Switzerland; 7 Department of Neurology, Laboratory of Neuroimmunology, Medical University of Lodz, 90-153 Lodz, Poland

**Keywords:** astrocyte, inflammation, chemokine, CXCL1, testosterone, androgen, demyelination, LPC, neuroprotection, remyelination

## Abstract

Astrocytes play an important role in the regulation of the inflammatory response in the CNS, e.g., in demyelinating diseases. Since the chemokine CXCL1 is known to be secreted by astrocytes and to have a pro-inflammatory effect on immune cells in the CNS, we verified the effect of testosterone on its secretion in vitro (in the astrocytic cell line DI TNC1). Testosterone reduced the increase in CXCL1 production caused by the pro-inflammatory agent lysophosphatidylcholine and restored the basal production level of CXCL1. The androgen receptor (present and functional in the studied cell line) was strongly suggested to mediate this effect—its non-steroid ligand flutamide exerted an agonist-like effect, mimicking the activity of testosterone itself on CXCL1 secretion. This novel mechanism has important implications for the known immunomodulatory effect of testosterone and potentially other androgenic hormones. It provides a potential explanation on the molecular level and shows that astrocytes are important players in inflammatory homeostasis in the CNS and its hormonal regulation. Therefore, it suggests new directions for the development of the therapeutic intervention.

## 1. Introduction

The inflammatory response in the central nervous system (CNS) is also known as neuroinflammation. This complex process is mediated by various cytokines and chemokines acting through specific receptors. Among the CNS resident cells, microglia and astrocytes are the most important sources of cytokines and chemokines. Astrocytes are considered a key element of neuroinflammation control by outside factors, including endocrine hormones. Following activation, astrocytes can become a potent source of proinflammatory cytokines such as TNFα, IL-12, IL-6, and IL-27, which are characteristic of the Th1 response. Upon a change of inflammatory milieu, astrocytes can also secrete the immunomodulatory cytokines IL-10 and IL-19, which are associated with the Th2 response [1,2,3,4,5,6]. Moreover, astrocytes secrete a repertoire of chemokines with different properties. Among them, CXCL1 is the most salient one, but others like CCL2, CCL3, and CXCL10 can also attract hematogenous cells such as T cells and macrophages, which may further enhance neuroinflammation. On the other hand, CXCL1, as well as CXCL2 and CXCL12, can promote repair by recruiting CNS resident cells such as oligodendrocyte progenitor cells (OPCs) [1,7,8,9].

We still do not understand well enough the mechanisms responsible for switching astrocyte function so that it promotes either damage to the CNS from excessive inflammation or its protection by anti-inflammatory mediator secretion. Deregulation of this response is strongly linked with the etiology of some demyelinating diseases of the CNS, including multiple sclerosis (MS) [1,10,11,12].

The chemokine (C-X-C motif) ligand 1 (CXCL1) is a small cytokine from the CXC chemokine family. CXCL1 is also known as melanoma growth-stimulating activity alpha (MGSA-α) and GRO-α in humans and as KC in mice [13]. Expression of CXCL1 has been observed in macrophages, neutrophils, epithelial cells, and glial cells [14,15,16]. Its effects are mediated through the chemokine receptor CXCR2 [16,17] and require binding to glycosaminoglycans (GAG) on endothelial and epithelial cells and the extracellular matrix [18]. High levels of CXCL1 are also able to stimulate the receptor CXCR1 [17].

When signaling through CXCR2, it bolsters the recruitment of neutrophils to the CNS. In other tissues, it is often a physiological response to microbial infection or tissue injury, but in the CNS, it is often pathological, e.g., in demyelinating diseases [19]. A recent report suggests that CXCL1 signaling is involved in microglia activation following brain injury [20]. While the role CXCL1 plays in OPC proliferation, migration, and differentiation into myelinating oligodendrocytes is multi-faceted [21,22,23,24], recent studies have identified mostly its negative effects, e.g., inhibition of the CXCL1/CXCR2 pathway, which promotes the differentiation of OPCs and consequently promotes myelin repair [25]. Astrocytes have also been reported to express CXCR2 receptors [26]. Since they are also a source of their ligand CXCL1, it suggests the possibility of an autocrine feedback loop regulating inflammation and remyelination.

Sex steroids influence not only the development and maintenance of reproductive systems but also several other organ systems, including the central nervous system (CNS) [27,28,29]. Among many functions, similarly to other steroid hormones, sex steroids (androgens, estrogens, and progestagens) exert several neuroprotective effects [30,31,32]. Within the context of immune response, particularly androgens and estrogens show effects on inflammatory cells and are potent modulators of immune responses within the CNS. It has become evident over last few decades that both the prevalence and severity of neuroinflammatory diseases of the brain and spinal cord are linked to sex hormones [33,34,35,36]. Epidemiological data from many studies show the higher prevalence of particularly demyelinating diseases of the CNS with neuroinflammation, such as MS, in women [37,38,39]. However, men with MS accumulate symptoms leading to a permanent neurological disability faster than female patients [40]. Moreover, men with testicular hypofunction are more likely to develop MS [41]. Testosterone is the major androgen with an important role in the physiology of both sexes. Testicles are the main source of testosterone in males; ovaries, adrenal glands, and adipocytes are the main source of this hormone in women. Testosterone and its metabolite dihydrotestosterone (DHT or 5αDHT) exert their principal function through a specific nuclear receptors—the androgen receptor (AR). Experimental data from animal models of MS further support androgens, particularly testosterone, as key players in alleviating inflammation-related pathological states in the CNS [42]. Experimental autoimmune encephalomyelitis (EAE) induced by the transfer of T cells shows a milder course when they are pre-treated with testosterone [43]. At the same time, testosterone has been shown to induce the production of anti-inflammatory IL-10 by T cells [44]. There is a large body of evidence pointing to resident and infiltrating immune cells as direct targets of androgen action in the CNS. However, despite the still-insufficient number of studies, the overall picture inexorably expands to include testosterone acting directly on astrocytes as well [45,46,47]. A key element of this mechanism is the recently confirmed expression of androgen receptors in astrocytes [48,49], but the functionality of this expression was hitherto in doubt.

In the present work, we present for the first time a direct mechanistic study of the axis androgen-astrocyte-chemokine in the context of inflammation. We tested the influence of primary androgen testosterone on CXCL1 expression and secretion, which turned out to depend strictly on the proinflammatory milieu. The use of the highly specific androgen receptor ligand flutamide, which is commonly applied as an antagonist of this nuclear receptor but has been previously shown to be able to have an agonist-like effect as well (which was also the case in our study), implicated this transcription factor in the observed phenomenon. Thus, we provide new insights on the molecular mechanism of this effect, which we found to most probably depend on the genomic action of the androgen receptor.

## 2. Materials and Methods

### 2.1. Cell Culture

Type 1 astrocyte cell line DI TNC1 was purchased from ATCC (cat. no. CRL-2005). The cells were cultured at 37 °C in a 5% CO_2_ humidified atmosphere in high glucose Dulbecco’s modified Eagle’s medium (Mediatech, Inc., Corning subsidiary, Manassas, VA 20109, USA, cat. no. 10-013-CV) supplemented with 10% (*v*/*v*) heat-inactivated fetal bovine serum (FBS) (Mediatech, Inc., Corning subsidiary, Woodland, CA 95776, USA, cat. no. 35-016-CV), 100 units/mL penicillin, and 100 μg/mL streptomycin (Mediatech, Inc., Corning subsidiary, Manassas, VA 20109, USA, cat. no. 30-002-CI).

### 2.2. Viability Assay

To verify the cytotoxic effect of lysophosphatidylcholine (LPC) treatment, the integrity of the cell plasma membrane was verified by staining cells with propidium iodide (cell impermeable) and Hoechst 33342 (cell permeable). Cells were seeded into a 96-well plate at a density of 1.0 × 10^4^ cells per well and treated with increasing concentrations of LPC (0−200 μg/mL) for 24 h. Following the incubation, cells were treated with 5 µM Hoecht33342 and 20 µM propidium iodide for 15 min at 37 °C. Subsequently, cells were washed once with phosphate-buffered saline (PBS, pH = 7.4) and fixed with 4% formaldehyde in PBS prepared freshly from paraformaldehyde. Cell viability was assessed with an automated fluorescence microscope, ArrayScan^®^ VTI (Thermo Fisher Scientific, Waltham, MA, USA), by comparing the number of cell nuclei stained with membrane-impermeable (propidium iodide) and permeable (Hoechst 33342) dye.

### 2.3. Gene Expression Assay

The gene expression level was determined by quantitative real-time RT-PCR. The aliquots of 2.6 × 10^5^ DI TNC1 cells were cultured for 24 h in 24-well plates in the presence or absence of 150 µg/mL LPC or with various concentrations of testosterone added from a stock solution prepared in ethanol. All cells, including control ones, were treated with the same amount of ethanol (0.2%). Following incubation, cells were washed once with PBS, pH = 7.4, and total cellular RNA was isolated using the InviTrap^®^ Spin Cell RNA Mini Kit (Stratec, Birkenfeld, Germany) according to the manufacturer’s protocol. Complementary DNA (cDNA) was transcribed from mRNA using the Maxima First Strand cDNA Synthesis Kit (Thermo Fisher Scientific, Waltham, MA, USA) and used for real-time PCR amplification with GoTaq^®^ qPCR 2x master mix (Promega Corporation, Madison, WI, USA) according to the manufacturer’s protocol. Each 16 μL reaction volume contained ca. 3 ng of cDNA and 0.25 μM of forward and reverse primers (for primer sequences, see Table 1). *Ywhaz*, *Ubc*, and *B2m* were used as reference genes. PCR reactions were performed in 96-well microplates using the CFX96 Real-Time PCR Detection System (Bio-Rad Laboratories, Hercules, CA, USA). The expression level of assayed genes was calculated using the ΔΔCt method and expressed as the number of mRNA copies per respective number of copies of geometric-averaged mRNA for reference genes.

### 2.4. Quantification of CXCL1 Secreted to the Medium by Astrocytes

At 72 h prior to medium collection, cells were seeded in 24-well plates at a concentration of 1.5 × 10^5^ cells per well. Cells were treated for 24 h with 150 µg/mL LPC and/or various concentrations of testosterone and/or flutamide prepared in fresh medium. Control cells were incubated with an equal concentration of ethanol (0.2%) that was used as a solvent for the respective treatments. At the time of medium collection, cells reached ca. 90% confluence and were counted to calculate the amount of CXCL1 secreted relative to 1.0 × 10^5^ of cells. Measurements were made with an enzyme-linked immunosorbent assay (ELISA) according to the manufacturer’s protocol (Rat CXCL1/CINC-1 DuoSet ELISA—R&D, cat. no. DY515). Cell-free medium was bound for 24 h to 96-well plates pre-coated with the capture antibody. After 2 h of incubation, the unbound material was washed off and a detection antibody was added for another 2 h incubation. Finally, the amount of bound antibody was detected colorimetrically, and the amount of CXCL1 was calculated from the calibration curve prepared for the recombinant protein included in the kit.

### 2.5. Statistical Analysis

The N values reported in the study refer to independent biological replicates. The Shapiro-Wilk test was used to evaluate whether the data follow a gaussian distribution. The effects of testosterone and flutamide on CXCL1 production by astrocytes were evaluated using three three-way ANOVA and, subsequently, Tukey’s post-hoc test to demonstrate the significance of differences between individual values. The evaluation of the effects of different doses of testosterone and flutamide was performed using the U Mann-Whitney test. Results of gene expression assays at the mRNA level were analyzed by the one-way ANOVA and, subsequently, Tukey’s post-hoc test to demonstrate the significance of differences between individual values and the control.

## 3. Results

### 3.1. Lysophosphatidylcholine Is Not Toxic to DI TNC1 Astrocytes but Exerts a Pro-Inflammatory Effect at the Signaling Level

Since incubation with the known in vivo inflammation stimulant lysophosphatidycholine (LPC) can change the composition of cell plasma membrane, leading to cytotoxicity, we verified the cytotoxic effect of LPC on DI TNC1 astrocytes. Cells were incubated with increasing concentrations of LPC, namely 0, 50, 100, 150, and 200 µg/mL, and the integrity of their cellular membrane was assessed after 24 h by comparing the amounts of cell nuclei stained with cell membrane permeable (Hoechst 33342) and impermeable (propidium iodide) dye. At all LPC concentrations, cell membrane integrity was preserved, since the number of cell nuclei stained by propidium iodide was less than 1% of those stained with Hoechst 33342.

**Figure 1:**
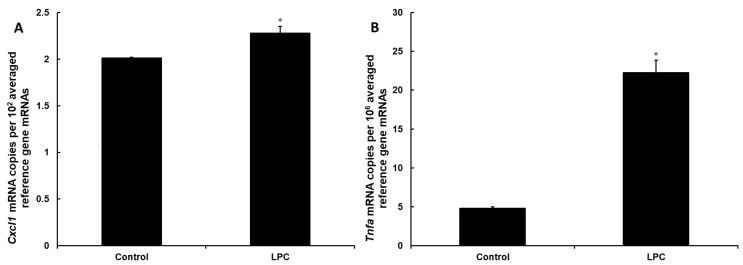
Incubation with LPC induces expression of proinflammatory genes in rat astrocytes. DI TNC1 cells were incubated for 24 h without (control) or in the presence of LPC (150 µg/mL). Subsequently, the expression of *Cxcl1* (**A**) and *Tnfa* (**B**) genes was quantified at mRNA level by real-time PCR, and expression level was expressed relative to a validated set of reference (housekeeping) genes. Significance of overall differences was tested by one-way ANOVA (*p* < 0.05). Significance of differences between treated samples and control was tested by Tukey’s post-hoc test (* *p* < 0.05).

Next, we tested if 24-h treatment with 150 µg/mL LPC has a pro-inflammatory effect on DI TNC1 astrocytes. At the mRNA level, the expression of the *Tnfa* gene increased by 360% (Figure 1A). At the same time, LPC treatment induced the expression of the *Cxcl1* gene by 13%, providing a mechanistic explanation for the increased secretion of this chemokine by cells incubated with LPC (Figure 1B).

### 3.2. DI TNC1 Astrocytes Produce an Increased Amount of CXCL1 When Treated with Lysophosphatidylcholine

**Figure 2:**
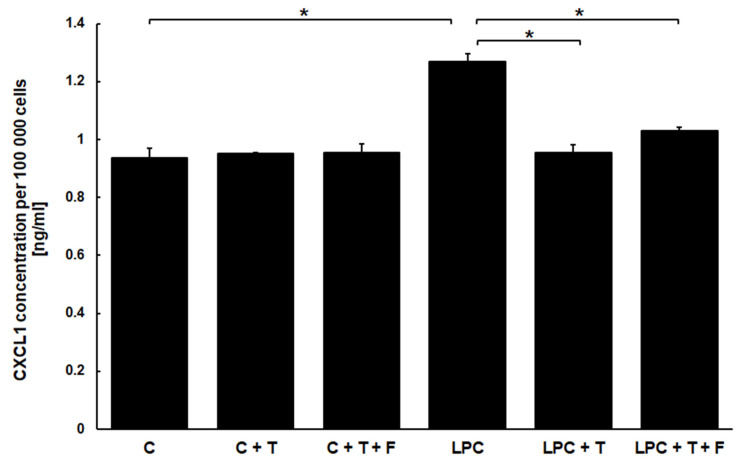
Quantification of CXCL1 secreted to culture medium by rat astrocytes. DI TNC1 cells were incubated for 24 h without (control, C), in the presence of LPC (150 µg/mL), in the presence of 60 µM testosterone (T), or in the presence of 60 µM testosterone and 60 µM flutamide (T + F). Subsequently, the amount of CXCL1 secreted by the cells was quantified in culture media by ELISA and it is presented as a value ± SEM per 100,000 cells. Significance of overall differences was tested by one-way ANOVA (*p* < 0.05). Significance of differences between treated samples and the control was tested by Tukey’s post-hoc test (* *p* <0.001), N = 4.

We have quantified the amount of CXCL1 that type I rat astrocytes (DI TNC1 cells) secreted into culture medium when incubated for 24 h with LPC (150 µg/mL) or in control conditions (cells treated with ethanol in equal volume used as a solvent for LPC). The level of CXCL1 in the culture medium was significantly increased in the group treated with LPC (Figure 2). The average concentration of CXCL1 in the culture medium was equal to 0.94 ± 0.03 ng/mL per 100,000 DI NTC1 cells. When stimulated with LPC, astrocytes produced 1.27 ± 0.03 ng/mL of CXCL1 per 100,000 DI NTC1 cells.

### 3.3. Testosterone Affects the Production of CXCL1 but Only in Cells Stimulated with Lysophosphatidylcholine

**Figure 3:**
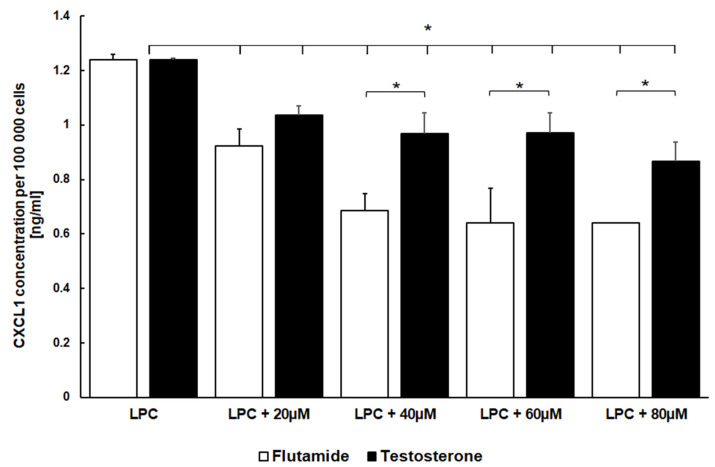
Quantification of CXCL1 secreted to culture medium by rat astrocytes in the presence of testosterone or flutamide. DI TNC1 cells were incubated for 24 h in the presence of LPC (150 µg/mL) and in the presence of increasing concentrations of either testosterone or flutamide. Subsequently, the amount of CXCL1 secreted by the cells was quantified in culture media by ELISA, and it is presented as a value ± SEM per 100,000 cells. * *p* < 0.05, N = 4.

Considering that incubation with LPC exerted a pro-inflammatory effect on DI TNC1 astrocytes and stimulated them to produce increased amounts of CXCL1, we were interested in verifying how testosterone affects this phenomenon. The amounts of CXCL1 secreted into culture medium by DI TNC1 astrocytes were quantified after 24 h of incubation with and without LPC and in the presence and absence of 60 µM testosterone (Figure 2). Testosterone antagonized the induction of CXCL1 stimulated by LPC treatment while having no effect on the basal level of secretion of this chemokine. Cells stimulated with LPC in the presence of testosterone and cells treated only with testosterone secreted CXCL1 in the same quantity as non-stimulated, control cells. Subsequently, we have set out to explore if this effect is dose-dependent. We have stimulated DI TNC1 astrocytes for 24 h with 150 µg/mL LPC in the presence of increasing concentrations of testosterone (0–80 µM). The concentration of secreted CXCL1 decreased gradually with an increasing dose of testosterone, reaching values similar to those secreted by the cells unstimulated with LPC (Figure 3). This observation confirms that testosterone mitigates the excess secretion of CXCL1 by binding to specific effectors (receptor-like effect), whose effectiveness is dependent on its concentration.

### 3.4. DI TNC1 Cells Express Functional Androgen Receptor

Subsequently, we have verified whether DI TNC1 cells are expressing functional androgen receptor (AR), which could be the target of action of testosterone in the studied phenomenon. First, we quantified the expression of the *Ar* gene in this cell line at the mRNA level by performing quantitative real-time PCR. We measured that the androgen receptor is expressed in DI TNC1 cells, and its expression was not affected by incubation with testosterone (data shown in Supplementary Materials—Tables S1–S4, Figure S1).

**Figure 4:**
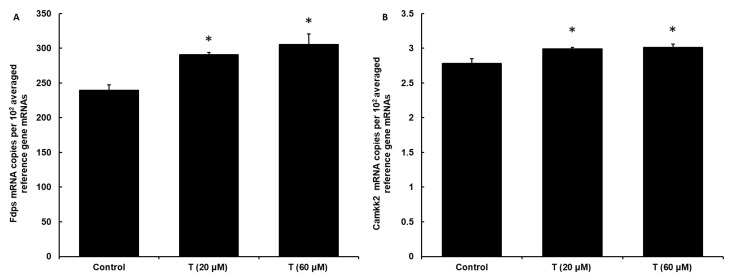
Incubation with testosterone induces expression of marker genes of androgen receptor in rat astrocytes. DI TNC1 cells were incubated for 24 h without (control) or in the presence of 20 µM or 60 µM testosterone. Subsequently, the expression of *Fdps* (**A**) and *Camkk2* (**B**) genes was quantified at mRNA level by real-time PCR, and expression level was expressed relative to a validated set of reference (housekeeping) genes. Significance of overall differences was tested by one-way ANOVA (*p* < 0.05). Significance of differences between treated samples, and control was tested by Tukey’s post-hoc test (* *p* < 0.05).

To assess if the effect of testosterone is mediated through the androgen receptor, we measured the expression of known AR-dependent marker genes: *Fdps* and *Camkk2* [52] upon stimulation with testosterone (Figure 4). Both 20 µM and 60 µM concentrations of testosterone enhanced the expression of the *Fdps* gene (by 21% and 27%, respectively) and the *Camkk2* gene (by around 10% at both concentrations), showing that the androgen receptor is stimulated by testosterone in DI TNC1 cells and implying that the effects of testosterone on CXCL1 secretion may be mediated through AR.

### 3.5. Flutamide Similarly to Testosterone Reduces the Pro-Inflammatory Stimulus of LPC on CXCL1 Secretion

To determine if the effect of testosterone on the secretion of CXCL1 is mediated by the androgen receptor, we incubated DI TNC1 cells for 24 h with testosterone and flutamide, which is usually used as an antagonist of testosterone’s effect on AR, in equal concentrations (60 µM). We tested its effects in the presence and absence of pro-inflammatory milieu (150 µg/mL of LPC). Cells unstimulated with LPC and incubated with both compounds secreted similar basal levels of CXCL1 (0.95 ± 0.03 ng/mL per 100,000 cells). Surprisingly, we observed that flutamide co-incubated with testosterone in the presence of LPC does not show antagonistic properties (Figure 2). Similar to cells treated with testosterone alone, the cells incubated with LPC in the presence of testosterone and flutamide produced almost the same amount of CXCL1 (1.03 ± 0.01 ng/mL per 100,000 cells).

To further corroborate this result, we investigated how different concentrations of flutamide (0–80 µM) affected the astrocytes in the presence of LPC (Figure 3). We observed the dose-dependent reduction of CXCL1 secretion by flutamide, but this effect was even more pronounced than the one observed with the same concentrations of testosterone, confirming that flutamide in this case probably exerts an agonistic effect on androgen receptors like testosterone, but even stronger.

## 4. Discussion

Inflammatory processes in the central and peripheral nervous systems, which accompany a variety of pathological states, are extremely complex phenomena that contribute to the etiology of clinically important neurological syndromes such as demyelinating and neurodegenerative diseases. To elucidate their mechanism, it is crucial to study the interplay and cross-communication between cells of the immune system and resident elements of the neural tissue, as both of these cell groups contribute to the distinctive elements of the inflammatory state. Chemokines play a pivotal role as messenger molecules. They can be secreted by non-specialized cells in order to signal specific immune cell populations to induce (or, in some situations, inhibit) their migration. They also have other properties, including activation, stimulation of the production of cytokines and other mediators, and specialized immune-related functions. There is a significant knowledge gap with regard to the involvement of individual cell types in producing the chemokine repertoire observed in neuroinflammation, for example concerning CXCL1, one of the most important chemokines with somewhat contradictory effects in the regulation of pathological inflammation. Research in this direction is needed, as this may be a potential therapeutic approach utilizing natural inhibitory mechanisms to counteract unwanted inflammation, which has deleterious consequences.

There is overwhelming evidence from animal models of neuroinflammation that CXCL1 production and secretion in the neural tissue are strictly regulated. It significantly impacts both the cellular and systemic presentation of pathological symptoms. Specifically, in some mouse models, the overexpression of CXCL1 under the glial fibrillary acidic protein (GFAP) promoter in astrocytes leads to a reduction in lesion load and enhances repair mechanisms in relapsing and remitting encephalomyelitis models [56]. Moreover, the chemokine receptor CXCR2 and its ligands CXCL1 and CXCL2 were shown to be upregulated during viral-induced demyelination, where CXCR2 signaling in oligodendrocytes seemed to play a role in their protection and the restriction of the demyelination process [57]. On the other hand, it has been shown that the rise in the expression of CXCL1 in astrocytes is associated with an increased severity of experimental autoimmune encephalomyelitis (EAE) due to the increased recruitment of neutrophils [58]. CXCL1 was reported to be upregulated during the acute phase of EAE, both in the brain and the spinal cord [8]. It has also been reported that CXCL1 was upregulated in mouse dorsal root ganglion (DRG) neurons during the asymptomatic phase of neuroinflammation. Neutrophils accumulated in the DRG produce neutrophil elastase, which is able to sensitize DRG neurons, leading to the induction of mechanical allodynia; therefore, gene silencing of CXCL1 attenuated neutrophil accumulation in the DRG and consequent mechanical allodynia [59]. Inhibition of CXCL1 signaling through CXCR2 by the use of anti-CXCR2 antibodies or pharmacological antagonists had beneficial effects for in vivo models of demyelination and encephalomyelitis, such as reduced size of lesions, increased OPC differentiation, functional improvement, enhanced myelination, and reduced lesion load. This was attributed to reduced infiltration and activation of macrophage/microglial cells under CXCR2 inhibition [60].

There are numerous natural mechanisms counteracting neuroinflammation at the level of cellular communication. Their potential therapeutic utilization is a hot topic in clinical studies, especially for demyelinating diseases. Among these, hormonal effects are very promising, including an emerging body of data on the favorable action of androgens in several in vivo models, which tie in, e.g., with epidemiological data on the penetration of demyelinating diseases in different sexes [38,61,62,63,64,65]. In the presence of testosterone, areas of LPC-induced focal demyelination in the spinal cord were repopulated with astrocytes to a much larger extent than in the absence of the hormone. The androgen-activated astrocytes promoted axonal remyelination through oligodendrocytes, whereas in control (untreated) lesions, Schwann cells were the main myelin-producing cell type [66]. This, along with other accumulated evidence for the anti-inflammatory action of testosterone in the CNS, is convincing at the physiological level in animal models; however, the specific molecular mechanisms at the cellular level and participating immune mediators need to be elucidated in direct biochemical experiments on isolated in vitro models, which is a prerequisite to understanding the feasibility of proposed modes of action. Therefore, the experiments presented in this paper fulfill an important role in providing basic data on how androgens can potentially counteract neuroinflammation. Our research demonstrates that they can directly (most probably via their nuclear receptor AR) suppress the stimulation of CXCL1 production in astrocytes by pro-inflammatory agents. Since CXCL1 is strongly implicated in enhancing the detrimental pro-inflammatory feedback loop by recruiting and activating neutrophils and/or macrophages, this suppression is one of the possible mechanisms explaining the moderating influence of testosterone on clinical manifestations of neuroinflammation.

While LPC is widely used in vivo as a demyelinating agent, it is also known to act as a bona fide proinflammatory mediator produced in the neural tissue itself [67,68]. It has been shown that LPC increases the production of pro-inflammatory cytokines and chemokines by immune cells [69,70]. In addition, LPC can also induce glial cell activation via the Rho-kinase pathway [71]. Enhanced expression of MCP-1 and CCR2 has been observed in activated microglia in response to LPC produced in astrocytes and neurons [72]. It has been observed that astrocytes and immature oligodendrocytes are sensitive to LPC-induced injury in vivo [68,73].

Our experimental model was composed of physiologically relevant elements, which enhanced the credibility of the proposed mechanism. Treatment with LPC mimics natural pro-inflammatory steps observed in demyelinating disease development pathways. We were able to further confirm this in our experiments showing the ability of LPC to induce the expression of another pro-inflammatory mediator, TNF-alpha. The DI TNC1 astrocyte cell line was derived from the same species and strain (Sprague-Dawley rats) and has been used in studies on experimental inflammatory demyelination, including those that identified astrocytes as potential mediators of androgen action. Our experimental approach involves measuring not only CXCL1 expression but, more importantly, its secretion to the outside environment, a central feature of the detrimental role of astrocytes in pro-inflammatory cell recruitment. Thus, the mechanism of action of the androgen-astrocyte-chemokine regulatory axis that emerges from our study can be directly applicable to in vivo models of neuroinflammation.

Taken together, our data points to a coherent mechanistic explanation of this regulatory phenomenon: Under the conditions of pro-inflammatory signaling (and only under these conditions), testosterone acts on the androgen receptor in astrocytes, preventing the signal-induced increase of CXCL1 secretion. In human monocytes exposed to parasitic (amoebal) antigens, CXCL1 secretion increased after androgen pre-treatment, which also points to the potential for androgen receptor-mediated modulation of the expression of this gene [74]. It is important to note that astrocytes produce and secrete a significant amount of CXCL1 in the resting state as well, and the molecular mechanisms responsible for this baseline level are not affected by testosterone. Identification of the exact elements (transcription factors) involved in CXCL1 expression in physiological and pathological conditions will require more profound exploration by molecular genetic techniques and is beyond the scope of the present study. However, the role of AR in the action of testosterone on astrocytes is strongly suggested by our experimental approach: we demonstrate that it is functionally expressed in the investigated cells (by showing the induction of known marker genes by testosterone [75]); testosterone acts in a dose-dependent (rather than threshold) manner on CXCL1 expression; and finally, and most convincingly, flutamide (which was applied as a presumptive antagonist of testosterone action) fortuitously turned out to have an analogous effect to testosterone in repressing CXCL1 induction. The latter argument points convincingly towards AR as the mediator of this effect, since there is a body of literature that identifies flutamide (or its metabolite, hydroxyflutamide) as a possible agonist of AR in several cell types [76,77,78]. This similarity between testosterone and flutamide is a powerful argument for the direct involvement of AR because there is no other known effector common to both of these chemically dissimilar ligands. It is, however, important to keep in mind that testosterone itself may indeed have other ways of inhibiting CXCL1 production in astrocytes, and therefore the effect of testosterone is potentially at least partially non-AR-dependent. One possible additional mechanism involves conversion to 17-beta-estradiol by aromatase expressed in these cells [79], with subsequent interference with the function of transcription factor AP-1, with which it is able to interact [80], and which is involved in CXCL1 induction during inflammatory signaling [81]. Importantly, estrogens have also been reported to increase CXCL-1 expression via estrogen receptor β (ERβ) [82]. Therefore, the conversion of testosterone to estrogen by aromatase could lead to a reduction of the original effect of testosterone, which could explain why flutamide alone demonstrated a stronger effect on CXCL1 level reduction than testosterone. In contrast, it has also been reported that estradiol at high concentrations down-regulates epithelial expression of CXCL1 [83]. In our study, we observed that the inhibitory effect of testosterone on CXCL1 production, while weaker at low doses, increases at the highest applied dose of 80 µM. This may be caused specifically by the shift in the effect of aromatase-produced estrogen at this concentration no longer counteracting the inhibitory effect of testosterone but enhancing it. Confirmation of the actual conversion of testosterone to estrogen occurring in astrocytes would help provide further proof for this postulate. Thus, further investigation of this mechanism is required to fully elucidate the mechanisms underlying the above effect. While we are certain that the AR-mediated pathway is involved in testosterone effects, further experiments are needed to quantify the extent of this involvement, e.g., using other AR antagonists or gene silencing.

The identification of a potential beneficial molecular mechanism of action of androgens in a model of neuroinflammation, with astrocytes as the novel site of action, is an important step in studies on hormonal regulation of CNS pathology. We demonstrate that (as was previously suggested on the basis of phenomenological in vivo data) testosterone and other androgens may indeed work via resident cells of the CNS which are not directly involved in immune activity and that astrocytes, which are already an important target in clinical studies on demyelinating and neurodegenerative diseases, acquire an even broader array of upstream regulators. This is also one of the first demonstrations of functional AR activity in astrocytic cells, while at the same time it adds to the increasing number of studies that encourage caution in the uncritical application of receptor antagonists as investigative and/or therapeutic tools since some of them (in this case flutamide) may also cause the opposite (agonistic) effect on some cell types. In general, the demonstration that pro-inflammatory chemokine secretion is an important function of astrocytes and that it can be modulated pharmacologically adds to our understanding of the complexity of neuroinflammation.

From a practical point of view, it is important to note that weak AR agonists, like testosterone and (hydroxy)flutamide, which may have beneficial effects in diseases that involve neuroinflammation, also have therapeutically favorable pharmacokinetics, being able to reach the CNS through the blood-brain and blood-cerebrospinal fluid barrier. Since CXCL1 has been implicated in many neuroinflammatory disorders and their models, such as EAE, MS, other demyelinating diseases, neurodegeneration, and infection, being able to modulate its secretion at one of its sources may be a common solution to seemingly unrelated pathologies. Of course, we do not suggest that this is the only chemokine that astrocytes use to influence the inflammatory milieu, nor do we suggest that androgens (or even AR itself) exert their documented beneficial physiological effects exclusively via the mechanism that we identified, but the fact that this signaling axis is theoretically possible in vivo is important for the interpretation of physiological phenomena such as sex differences in disease penetration or hormonal effects in MS. From the clinical point of view, another important corollary is the possibility of side effects of hormonal treatments in the CNS. However, the potentially most important therapeutic implication of our results is the indication that astrocytes are a viable target for AR-mediated adjuvant treatment of demyelinating diseases by alleviating the pathologically increased production of at least some pro-inflammatory mediators.

In the future, it will be important to verify whether AR binds directly to the CXCL1 promoter or whether its action is indirect. We also plan to work on identifying the physiologically important cellular targets of astrocyte-derived CXCL1 (resident vs. hematogenous cells) and optimizing the agonistic function of androgens in animal models.

In conclusion, the probable direct involvement of AR in the effects of androgen on the pro-inflammatory activity of astrocytes is a newly identified mechanism of hormonal regulation of neuroinflammation. This mechanism involves the inhibition of CXCL1 release, underlining the central role this chemokine plays in regulatory loops between glial and immune cells in the CNS.

## Figures and Tables

**Table 1:** Sequences of primers used in this study.

Gene	Forward and Reverse Sequences (5′-3′)	Source
*Ywhaz*	Fw: AACTTGACATTGTGGACATCGG Rv: AAAGGTTGGAAGGCCGGTTA	this study
*Ubc*	Fw: ACACCAAGAAGGTCAAACAGGA Rv: CACCTCCCCATCAAACCCAA	[50]
*B2m*	Fw: GTCACCTGGGACCGAGACAT Rv: AGAAGATGGTGTGCTCATTGC	[51]
*Ar*	Fw: CTTATGGGGACATGCGTTTGG Rv: GCTCCGTAGTGACAACCAGA	this study
*Fdps*	Fw: GCAGACTCTCGACCTCATCACA Rv: CCCATCAATTCCAGCCATG	[52]
*Camkk2*	Fw: AGAACTGCACACTGGTCGAG Rv: CCGGCTACCTTCAAATGGGT	[53]
*Cxcl1*	Fw: GCCACACTCAAGAATGGTCG Rv: TGGGGACACCCTTTAGCATC	[54]
*Tnfa*	Fw: GACCCTCACACTCAGATCATCTTCT Rv: TGCTACGACGTGGGCTACG	[55]

## Data Availability

The original contributions presented in the study are included in the article/Supplementary Materials, further inquiries can be directed to the corresponding author/s.

## Supplementary Materials

The following supporting information can be downloaded at: https://www.mdpi.com/article/10.3390/cimb46030135/s1. Tables S1–S4: Analysis of expression of the *Ar* gene in DI TNC1 cell line using quantitative real-time PCR. Figure S1: The expression of *Ar* gene in DI TNC1 cell line.
