# Supplementary material for: Testosterone Inhibits Secretion of the Pro-Inflammatory Chemokine CXCL1 from Astrocytes
Source: Curr Issues Mol Biol. 2024 Mar 6;46(3):2105–18. doi: 10.3390/cimb46030135 (PMC10968986; doi:10.3390/cimb46030135)
Supplement: Supplementary file 1 [file cimb-46-00135-s001.zip › cimb-2545094-supplementary.pdf]

Supplementary materials.

Tables S1-4. Analysis of expression of the *Ar* gene in DI TNC1 cell line using quantitative real-time PCR.

Table S1.

Ct	Ywhaz_rat_1	Ywhaz_rat_2	B2M_rat_1	UBC_rat_1	**AR_3**
RNA control	N/A	39,06	N/A	35,03	**40,87**
	N/A	38,55	N/A	34,51	**44,18**
DI TNC1 control	17,32	18	16,45	17,44	**28,07**
	17,25	17,99	16,55	17,4	**28,58**
DI TNC1 testosterone 5µM	16,88	17,9	16,35	17,71	**28,77**
	17,18	17,99	16,31	17,52	**28,42**
DI TNC1 testosterone 40µM	17,15	17,95	16,18	17,35	**28,23**
	16,73	17,94	16,24	17,34	**28,37**

Table S2. Mean:

Ct	Ywhaz_rat_1	Ywhaz_rat_2	B2M_rat_1	UBC_rat_1	**AR_3**
ctrl	17,285	17,995	16,5	17,42	**28,325**
testo 5µM	17,03	17,945	16,33	17,615	**28,595**
testo 40µM	16,94	17,945	16,21	17,345	**28,3**

Table S3.

**geo mean HKG**	
ctrl	17,29
testo 5µM	17,28
testo 40µM	17,15

Table S4.

***Ar* mRNA copies per 10^4 averaged reference gene mRNAs**	
C	4,78
T 5µM	3,93
T 40µM	4,40

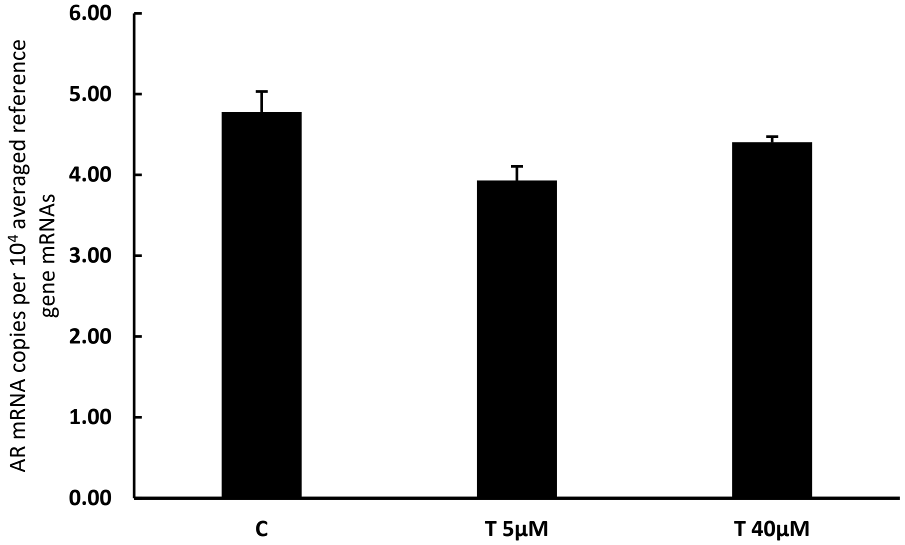

Figure S1: The expression of *Ar* gene was quantified at mRNA level by real-time PCR and expression level was expressed relative to a validated set of reference (housekeeping) genes. It was confirmed that DI TNC1 cell line expresses androgen receptor. Significance of overall differences was tested by one-way ANOVA ($p<0.05$). Significance of differences between treated samples and control was tested by Tukey's post-hoc test (* - $p <0.05$).
